# A Surface Electromyography (sEMG) System Applied for Grip Force Monitoring

**DOI:** 10.3390/s24123818

**Published:** 2024-06-13

**Authors:** Dantong Wu, Peng Tian, Shuai Zhang, Qihang Wang, Kang Yu, Yunfeng Wang, Zhixing Gao, Lin Huang, Xiangyu Li, Xingchen Zhai, Meng Tian, Chengjun Huang, Haiying Zhang, Jun Zhang

**Affiliations:** 1 Institute of Microelectronics of the Chinese Academy of Sciences, Beijing 100029, China; wudantong@ime.ac.cn (D.W.); tianpeng@ime.ac.cn (P.T.); zhangshuai@ime.ac.cn (S.Z.); wangqihang@ime.ac.cn (Q.W.); yukang@ime.ac.cn (K.Y.); wangyunfeng@ime.ac.cn (Y.W.); gaozhixing@ime.ac.cn (Z.G.); huanglin@ime.ac.cn (L.H.); lixiangyu@ime.ac.cn (X.L.); zhaixingchen@ime.ac.cn (X.Z.); tianmeng@ime.ac.cn (M.T.); huangchengjun@ime.ac.cn (C.H.); 2 University of Chinese Academy of Sciences, Beijing 100049, China

**Keywords:** surface electromyography, wearable system, muscle status monitoring, grip force prediction

## Abstract

Muscles play an indispensable role in human life. Surface electromyography (sEMG), as a non-invasive method, is crucial for monitoring muscle status. It is characterized by its real-time, portable nature and is extensively utilized in sports and rehabilitation sciences. This study proposed a wireless acquisition system based on multi-channel sEMG for objective monitoring of grip force. The system consists of an sEMG acquisition module containing four-channel discrete terminals and a host computer receiver module, using Bluetooth wireless transmission. The system is portable, wearable, low-cost, and easy to operate. Leveraging the system, an experiment for grip force prediction was designed, employing the bald eagle search (BES) algorithm to enhance the Random Forest (RF) algorithm. This approach established a grip force prediction model based on dual-channel sEMG signals. As tested, the performance of acquisition terminal proceeded as follows: the gain was up to 1125 times, and the common mode rejection ratio (CMRR) remained high in the sEMG signal band range (96.94 dB (100 Hz), 84.12 dB (500 Hz)), while the performance of the grip force prediction algorithm had an R^2^ of 0.9215, an MAE of 1.0637, and an MSE of 1.7479. The proposed system demonstrates excellent performance in real-time signal acquisition and grip force prediction, proving to be an effective muscle status monitoring tool for rehabilitation, training, disease condition surveillance and scientific fitness applications.

## 1. Introduction

Skeletal muscle, as a fundamental component of the human body, plays an indispensable role in sustaining life activities. With changes in lifestyle, aging [1], or the impact of diseases and injuries [2,3], skeletal muscles may gradually lose strength and endurance, leading to a decline in mobility, which can significantly affect an individual’s daily life and overall health. Interventions such as exercise training [4,5] and rehabilitation therapy [6,7] have been proven to effectively improve skeletal muscle function.

However, the effectiveness of these external interventions largely depends on the accurate monitoring and assessment of muscle status. Muscle status refers to a series of indicators reflecting muscle health and function, including, but not limited to, muscle force, coordination, fatigue, and pathological status. Due to individual differences and the complexity of muscle conditions, it is difficult to precisely control the intensity of external interventions, which may lead to suboptimal outcomes or even adverse consequences. Therefore, the ability to monitor muscle status in real-time and accurately, as well as to adjust the intensity of interventions based on the monitoring results (achieving a closed-loop control between muscle status monitoring and external interventions), has become a hot topic in current research. This is particularly significant in applications such as remote rehabilitation [8] and autonomous scientific exercise in unsupervised environments.

Common muscle status monitoring technologies include the following: dynamometers [9], near-infrared spectroscopy (NIRS) [10], ultrasound imaging [11,12], magnetic resonance imaging (MRI) [13], electrical impedance myography(EIM) [14,15], and surface electromyography (sEMG) [16]. SEMG, a non-invasive technique measured on the skin surface, reflects the cumulative effect of muscle action potentials and neural activities on the skin [16], providing valuable information about neuromuscular activities [17]. Compared to other monitoring technologies, sEMG is portable and capable of real-time feedback, allowing for dynamic measurements and directly reflecting the muscle contraction state that causes limb movement. This makes it a key source of feedback for external intervention measures and holds significant research importance, making it a popular research tool in sports and rehabilitation sciences.

Muscle force monitoring is a crucial aspect of evaluating the body’s muscular health and guiding rehabilitation therapy. There is a robust correlation between sEMG technique and muscle force. The Hill-type muscle model establishes the relationship between muscle contraction and force [18], and muscle contraction force exhibits an exponential relationship with EMG signals [19]. David G. Lloyd and Thor F. Besier used sEMG signals to obtain muscle activation and calculated muscle force by combining the modified Hill model with activation and tendon length [20]. Based on these correlations, there has been considerable research on force prediction using sEMG in recent years, with studies utilizing sEMG signals to predict grip force [21] and grasping force [22], which are applied in rehabilitation process assessment and fine control of prosthetics. Grip force measurement is a straightforward, easy, and non-invasive method of assessing overall muscle force. It has been demonstrated that grip force can be employed as a reliable indicator of muscle weakness in the upper limbs during the chronic post-stroke period [23]. Additionally, sEMG can accurately reflect local muscle fatigue in the human body [24] and is an effective way of tracking and evaluating muscle coordination [25], predicting forearm movements [26] and joint angle [27].

Recently, researchers have been effectively evaluating muscle status by using commercial devices or self-developed sEMG acquisition devices. Adyasha Dash and Uttama Lahiri developed a virtual-reality-based sEMG-triggered grip exercise platform called Gripx using Biopac MP150 (Biopac Systems Inc., Goleta, CA, USA). They quantified and graded grip strength through sEMG signal assessment of muscle activation and provided visual feedback. The results demonstrated huge potential for grip recovery [28]. Xu and colleagues used Delsys (Delsys Inc., Natick, MA, USA) to collect sEMG and classified four natural grasping actions with an SVM algorithm, achieving an average accuracy rate of 91.43% to 97.33%. They estimated the corresponding force of the actions using a back propagation (BP) neural network, with an average R^2^ of 0.9082, which aids in the natural control of myoelectric prosthetics and the application of EMG-based rehabilitation training systems [29]. Zhao and others proposed a wearable monitoring device for upper limb rehabilitation, integrating ECG and sEMG sensors. The results showed that monitoring ECG and EMG signals can help patients enhance upper limb improvement according to treatment settings and user needs [30]. Chiako Mokri and colleagues designed a knee joint rehabilitation robot based on sEMG signal force estimation. They studied and estimated the exerted force using wired sEMG signals and found that a support vector regression (SVR) model optimized by a genetic algorithm (GA) provided the best performance [31]. Saara M. Rissanen et al. designed a wearable device for assessing daily motor fluctuations in Parkinson’s disease patients by dynamically monitoring their surface electromyography (sEMG) and accelerometer (ACC) signals on a 24 h basis, finding that the monitoring method significantly correlated with symptomatic changes in patients’ clinical scores and home diary entries, which could help to adjust medication and deep brain stimulation (DBS) therapy [32]. Fialkoff, B. and others proposed a new method for generating inverse EMG images of the forearm based on functional potentials, using sEMG signals collected uniformly around the forearm with eight electrodes [33]. Li and the team designed a simple-structured, cost-effective eight-channel wearable surface EMG acquisition system, including a signal collection part and a receiving part. The system demonstrated good capabilities in collecting sEMG signals [34].

In this work, we primarily proposed and designed a multi-channel sEMG acquisition system for objective monitoring of the grip force. The system includes an sEMG acquisition module and a host computer receiving module, and it supports four-channel data acquisition, processing, and wireless transmission. An isometric grip strength contraction experiment is designed to collect the data of sEMG signals and grip strength. According to the collected data, a grip force prediction model is established based on the bald eagle search optimization (BES) and random forest (RF) algorithms. In the future, this system allows users to receive objective muscle status information, enabling more effective guidance for external intervention measures. This work is expected to provide a more convenient and effective monitoring method for the rehabilitation of patients, condition monitoring for Parkinson’s patients, or the scientific exercise of healthy individuals.

## 2. Methods

**Figure 1:**
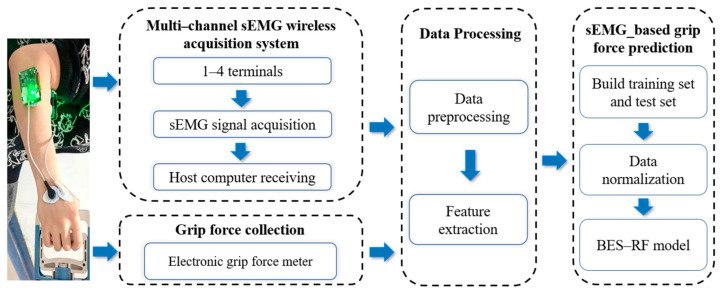
Workflow of this paper.

In this study, a multi-channel sEMG wireless acquisition system was designed, and it can support four-channel sEMG-signal wireless transmissions through four signal acquisition terminals. Experimental data collection was performed using an electronic grip force meter and the acquisition system described above. The magnitude of grip force values and the sEMG signals of two forearm muscles were collected from each volunteer at different grip forces, respectively. Then, the sEMG signals were pre-processed and feature extracted before, finally, the BES−RF algorithm was designed to build a grip force prediction model. Figure 1 illustrates the workflow of this paper.

### 2.1. Multi-Channel sEMG Wireless Acquisition System Design

#### 2.1.1. The Overall System Framework

**Figure 2:**
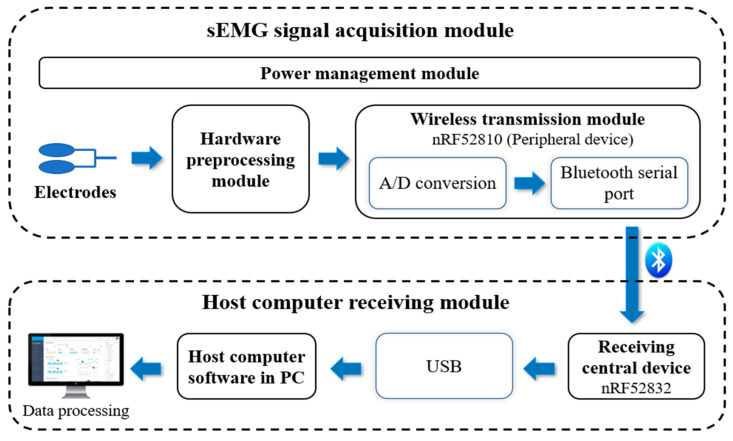
Block diagram of multi-channel wireless sEMG acquisition system.

**Figure 3:**
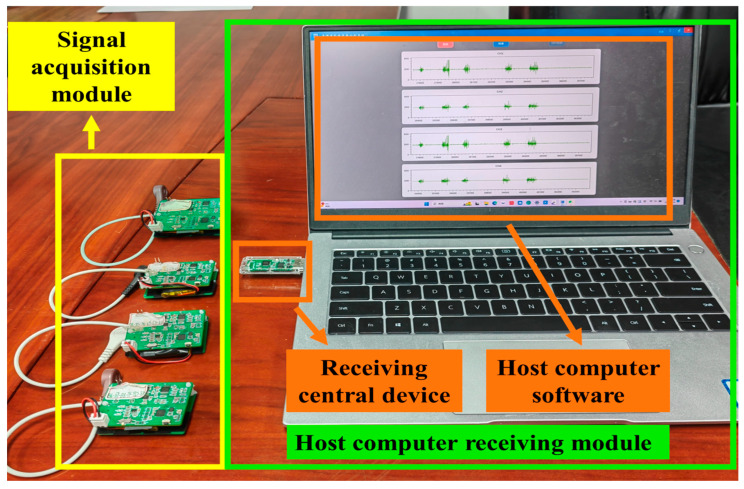
Physical diagram of multi-channel wireless sEMG acquisition system.

The sEMG has an amplitude range in the µV level and a frequency range between 20 and 500 Hz [35,36], which makes it susceptible to noise interference. Therefore, the processing circuit must fulfill the requirements of high input impedance, high gain, high CMRR, and low noise [37,38]. Most existing studies have used wired transmission to acquire sEMG [33,39,40], which can limit the user’s range of motion and inconvenience remote rehabilitation monitoring. In contrast, wireless acquisition allows users to move freely and realize remote dynamic monitoring. Most of the existing wireless sEMG systems (NORAXON and Delsys in the USA, MEGA in Finland, etc.) are not suitable for a wide range of applications due to their high cost, large size, and poor compatibility. To address the above problems, a multi-channel wireless sEMG acquisition system is designed in this study. The system block diagram is shown in Figure 2 and the physical system diagram is shown in Figure 3. The system contains a signal acquisition module and a host computer receiving module. The sEMG signal acquisition module consists of EMG electrodes, a hardware pre-processing module, a wireless transmission module, and a power management module. The system is controlled by the MCU integrated into the wireless transmission module. The host computer receiving module consists of the receiving central device and the host computer software.

#### 2.1.2. Acquisition Module Design

**Figure 4:**
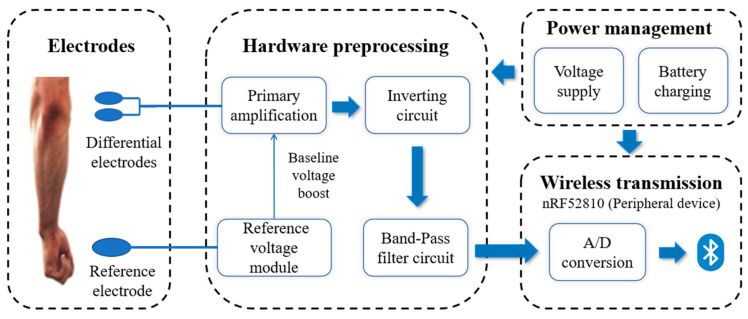
The block diagram of the sEMG signal acquisition module.

The block diagram of the acquisition module is shown in Figure 4, including the EMG electrodes, hardware pre-processing module, wireless transmission module, and power management module.

The circuit structure was designed with miniaturization for the needs of portable systems. Differential Ag/AgCl conductive gel electrodes were utilized to acquire the sEMG signals with an electrode spacing of 3 cm. Differential electrodes will effectively suppress common mode interference. Snap buttons were selected to connect the collection electrodes to the preamplifier circuit with the shortest distance. When drawing the circuit board, symmetrical differential signals can effectively suppress the differential mode interference brought about by the asymmetry of the alignment. The reference electrode was used to reduce the industrial frequency electromagnetic interference. The differential signals were transferred to the hardware pre-processing module for processing. The signal was implemented with primary amplification through an AD8227 instrumentation amplifier (ADI, Wilmington, MA, USA), whose high common mode rejection ratio (CMRR) effectively suppresses common-mode interference in the circuit, and a resistor was set up to achieve 45-fold primary signal amplification. An inverting circuit module was designed using AD8646 (ADI, MA, USA) to invert the output of the preamplifier circuit once. This further suppressed the common-mode voltage, resulting in a higher output signal-to-noise ratio. The baseline of the sensor circuit was added to 1/2 VCC by the reference voltage module, and the EMG signal, which is alternating in nature (contains negative values), was added above 0 V to facilitate A/D conversion. The signal was then passed through an AD8648 (ADI, MA, USA) filter circuit to achieve bandpass filtering from 15.4 to 1.86 kHz. The secondary amplification was set to 25×, ultimately achieving a two-stage amplification of 1125×.

The hardware pre-processed signals were then processed in the nRF52810 (Nordic Semiconductor, Trondheim, Norway), a low-power Bluetooth SoC that incorporates Bluetooth, an ADC, and an MCU containing other peripherals. The four sEMG signals entered the on-chip ADC for continuous signal conversion. The ADC has a resolution of 12 bits, meaning it can detect 4096 analog levels. The converted digital signals were transmitted wirelessly via the on-chip Bluetooth. nRF52810 was the peripheral device. Thus, the following data transfer protocol was set up to transmit data to the central device: sending one packet of data every 50 ms, with 20 packets being sent in 1 s. The length of each packet was 116 bytes, including packet header (2 bytes), frame header (4 bytes), MAC address (6 bytes), 50 myoelectric data (100 bytes), end-of-data flag (2 bytes), and packet end (2 bytes). Eventually, we achieved a synchronized transmission of signals from 1 to 4 channels with a sampling rate of 1000 Hz. Each acquisition terminal had its own unique MAC address.

A 3.7 V Li-ion battery was selected to supply power for our system. TP4056M (Top Power ASIC, Nanjing, China) was used to manage the charging of the Li-ion battery.

**Figure 5:**
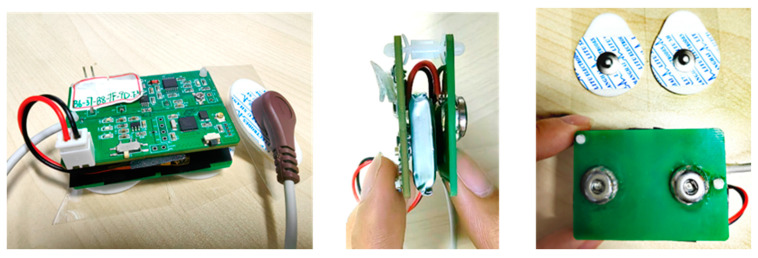
The physical structure of an acquisition terminal, shows the front, side and back.

The physical structure of an acquisition terminal is shown in Figure 5.

#### 2.1.3. Design of the Host Computer Receiving Module

The signals from the acquisition terminal were received through the receiving central device, nRF52832 (Nordic Semiconductor) and passed to the PC for processing through the universal serial bus (USB). We designed a host computer software for data acquisition and pre-processing. The upper computer software was developed based on the .Net Core6.0 framework and adopted the Windows Presentation Foundation (WPF) user interface framework and Prism Mvvm mode, etc. The acquisition interface includes functions such as modularization, separation of business logic, and an acquisition interface, and it can receive 4-channel sEMG data and display real-time waveforms via a COM interface. Using the SQLite database as a local data persistence solution, we can save data for individuals and export data at any time.

### 2.2. Data Acquisition

In this work, six healthy subjects were recruited, including males and females, from a 20 to 30 age group. None of the subjects had any history of neurological or muscular disorders. Written informed consent was provided to all subjects. Each subject was asked to read and sign the informed consent form.

Grip force isometric muscle contraction experiments were designed for inquiry as a proof-of-concept experiment. To measure the data, the grip force meter and the self-developed multi-channel sEMG wireless acquisition system were employed in the processes. The EH101 electronic grip force meter (Xiangshan, China) was selected due to its accurate measurement, reliable quality, simple operation, and convenient reading. A grip span of 55 mm was chosen for the grip force meter [41].

**Figure 6:**
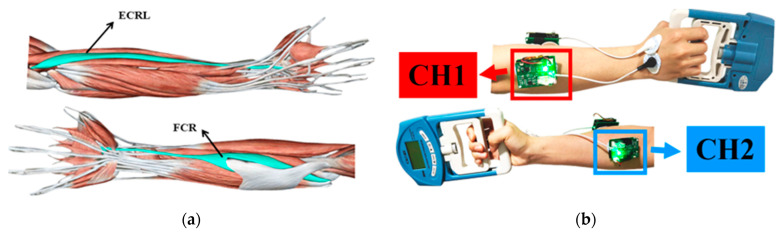
Muscle and terminals attachment locations: (**a**) shows the location of ECRL muscle and FCR muscle; (**b**) shows the attachment location of the two terminals (CH1 was attached to the ECRL and CH2 was attached to the FCR).

Maximum voluntary contraction (MVC) is a measurement of maximum muscle strength and is used to evaluate individual muscular functions and set the basic strength training. In order to make the muscle information of all subjects comparable, the experiment used the relative grip force standardized by the subject’s MVC. Grip force and sEMG signals at MVC were collected and recorded separately for each subject before the start of the experiment. Subsequently, the magnitude of grip force values at 80%, 60%, and 40% MVC and the sEMG signals of the two forearm muscles were collected and recorded simultaneously for each volunteer. Since the sEMG signal acquisition requires the conductive electrodes to be attached to the skin, the superficial forearm muscles that are under the skin were selected (Figure 6a). i.e., the extensor carpi radialis longus muscle (ECRL) and flexor carpi radialis muscle (FCR).

The following steps refer to the SENIAM guidelines [42]. After removing body hair, cleansing the skin with medical alcohol, and lightly scrubbing the skin, the surface electrodes were attached to the muscle belly of the muscle to be tested. The surface electrodes oriented as far as possible in the direction of the longitudinal axis of the muscle fibers. In general, due to possible differences in muscle activity patterns and electrode positions among individuals, it is necessary to place the reference electrode in a location that is far away from the target muscle, has the least risk of large common mode interference signals, and is relatively stable and minimally affected by movement. In accordance with the SENIAM guidelines and research [43], the dorsal side of the wrist was chosen as the reference electrode position for measuring sEMG signals from the forearm muscles. CH1 was attached to the ECRL and CH2 was attached to the FCR, as shown in Figure 6b. Verification of surface electromyography (sEMG) signal quality was achieved by observing the waveforms on the host computer after sensor placement.

**Figure 7:**
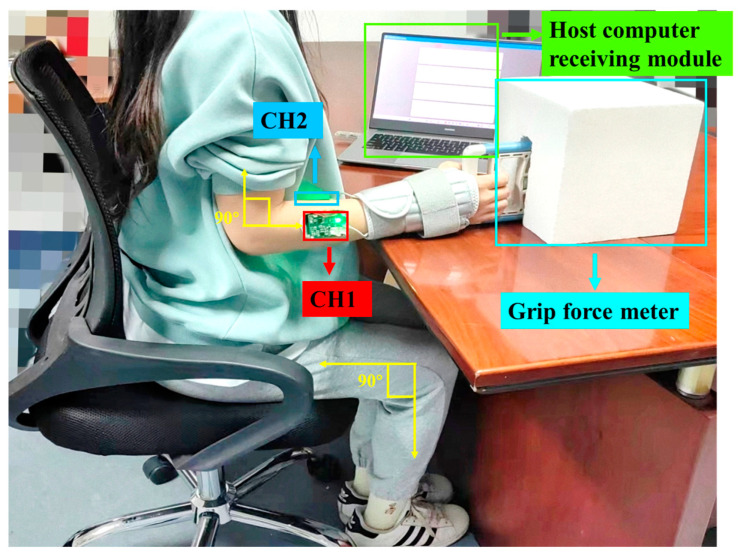
Demonstration of actual data acquisition scenarios.

In accordance with the standardized guidelines for the measurement of grip force put forward by the ASHT in 1992, the subjects were placed in a sitting position to carry out the test. The subject’s shoulder joint was positioned in internal retraction and neutral rotation, and the arm was securely attached to the body through a combination of experimental document reading instructions and real-time supervised observation. The subject’s forearm was positioned close to the table in a neutral position, with elbows being placed at a 90° angle by adjusting the height of the seat. Additionally, a wrist fixation brace was used to immobilize the subject’s wrist in a neutral position. The grip force meter was attached to the tabletop using a specially designed device. Subjects were required to perform each movement for not less than 2 s and to rest for 20 s at the end of each movement to prevent muscle fatigue. The subjects were required to remain motionless during the experiment and were allowed to move slightly unless the experimenter asked them to relax. The actual data acquisition experimental process scenarios are shown in Figure 7.

### 2.3. Data Pre-Processing

Because the EMG signal is a non-stationary and weak signal, it is susceptible to noise and has an alternating nature, and so the raw signals between different subjects are not comparable. In order to facilitate the subsequent work, the data need to be pre-processed. 

The EMG signal pre-processing process includes multi-channel signal reconstruction, segmentation, filtering, rectification, and normalization [31,44], which is based on the implementation of MATLAB.

**Figure 8:**
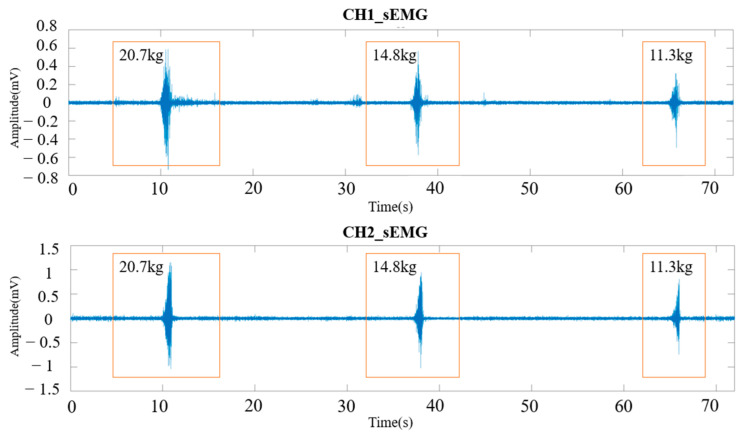
sEMG signals after reconstruction and reference voltage removement with corresponding grip force.

The received sEMG data were stored by the host computer according to the data transfer protocol mentioned in Section 2.1.2. The two channels of the sEMG signal were reconstructed on the PC side by classifying the MAC address and combining the valid sEMG data bytes in packet header order. The reference voltage was removed while reconstructing to return the baseline from 1/2 VCC to 0 V. The data were then segmented, and each valid segment of the sEMG signal was saved by manually calibrating the start and end points. The sEMG signals after this process and corresponding grip force are shown in Figure 8.

**Figure 9:**
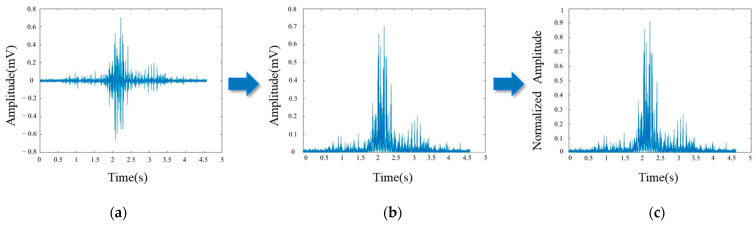
Data pre-processing: (**a**) the single sEMG signal after data segmentation; (**b**) the sEMG signal after band-pass filtering and full-wave rectification; (**c**) the sEMG signal after MVC normalization.

Then, data segmentation was performed to save each segment of valid sEMG signals. Subsequently, each segment of the EMG signal was band-pass filtered using a zero-phase fourth-order Barwaters filter with a frequency range of 20–490 Hz, which is the fundamental frequency range in which the sEMG signal is located. Due to the alternating nature of the EMG signals, full-wave rectification was required after the filtering was completed to flip the negative values above the baseline. Finally, the maximum value of MVC of each volunteer was made as the standard, respectively, and each data point was normalized according to Equation (1). The process is shown in Figure 9.

(1) $${\mathrm{sEMG}}_{\mathrm{nor}}=\left(\frac{{\mathrm{sEMG}}_{\mathrm{task}}}{{\mathrm{sEMG}}_{\mathrm{MVC}}}\right)\times 100\%$$

where sEMG_nor_ is the normalized sEMG signal value, sEMG_task_ is the current raw EMG signal of this subject, and sEMG_MVC_ denotes the maximum value of the sEMG signal of this subject at maximum voluntary contraction force.

### 2.4. Feature Extraction

Since the sEMG signal contains too much information, if the above pre-processed sEMG signal is directly used as the input of the model, there will be more interference, leading to the deterioration of the model performance; therefore, feature extraction is of great importance in EMG signal processing [39]. The sliding window method was chosen for feature extraction of EMG signals to improve the utilization of EMG signals. The effects of several window lengths were compared in [33,45,46], and it was found that the window length of 256 ms showed the best performance, so the window length of 256 ms and the step size of 56 ms were chosen in this study.

The features often extracted for analyzing sEMG signals include time–domain, frequency–domain and time–frequency–domain features. In this paper, some of the more commonly used time and frequency domain features are extracted for regression analysis of grip force. The time–domain features include integrated electromyographic value (iEMG), root mean square (RMS), waveform length (WL), and Shannon entropy (SE). Frequency domain features include median frequency (MF) and mean power frequency (MPF).

The iEMG is the sum of the area surrounded by the curve per unit time after the measured sEMG signal is rectified and smoothed.

(2) $$\mathrm{iEMG}=\frac{1}{N}\sum_{i=1}^{N}\left|x_{i}\right|$$

where x_i_ {1 ≤ i ≤ N} is used to denote the ith point in an sEMG window. N is the length of the window.

RMS is the square root of the mean value of the amplitude of the sEMG signal over a certain time window and is commonly used to assess the strength and fatigue of muscle contractions.

(3) $$\mathrm{RMS}=\sqrt{\frac{1}{N}\;\sum_{i=1\;}^{N}x_{i}^{2}}$$

WL is the cumulative sum of the waveform lengths of the sEMG signals within a certain time window. It is commonly used to assess the contraction and relaxation states of muscles.

(4) $$\mathrm{WL}=\sum_{i=1}^{N}\left|x_{i+1}\;-\;x_{i}\right|$$

SE is used to measure the complexity of a signal sequence. The more complex the signal sequence, the larger it is. SE is calculated as:

(5) $$H(X)=-\;\sum p\left(x_{i}\right)\;\times \log_{2}p(x_{i})$$

H(X) denotes the information entropy of the source X and p(x_i_) denotes the probability of a symbol x_i_ in the source X.

MF is the median frequency of the electromyographic signals generated during muscle contraction and is commonly used to assess the force and fatigue of a muscle contraction. MF is derived by analyzing the power spectral density (PSD) of the surface EMG signal.

(6) $$\mathrm{MF}=\frac{1}{2}\int_{0}^{\infty }\mathrm{PSD}\left(f\right)\mathrm{df}$$

MPF is the average of the power frequency distribution of EMG signals generated during muscle contraction, which can be used to assess the strength and fatigue of muscle contraction.

(7) $$\mathrm{MPF}=\frac{\int_{0}^{\infty }f\cdot \mathrm{PSD}\left(f\right)\mathrm{df}}{\int_{0}^{\infty }\mathrm{PSD}\left(f\right)\mathrm{df}}$$

### 2.5. Grip Force Prediction

There are commonly two methods used for analyzing the relationship between sEMG signals and grip force: model-based methods and machine learning methods. The former obtains the final grip force by establishing the relationship between the sEMG signal, muscle model, and muscle output force; the latter uses the sEMG signal as the input and the force signal as the output to establish the fitting relationship. In this study, we choose to use the Random Forest algorithm in machine learning and try to use the bald eagle search optimization (BES) algorithm for parameter optimization of RF to predict the grip force.

#### 2.5.1. Random Forest Regression

Intelligent algorithms are developing very rapidly, and algorithms such as ANN, BP neural network, SVR, RF, etc. are widely used in the fields of gesture recognition, prosthesis control, joint angle prediction, and so on. This work investigates grip force prediction based on sEMG signals. It was found that RF and CNN models performed best in predicting joint kinematics and muscle strength within and between subjects due to their providing the smallest prediction error and requiring the least amount of computational time for training and testing [47]. Therefore, after acquiring and processing the sEMG signal and the hand grip force signal, we chose to train the grip force prediction model using RF. Random Forest (RF) was proposed by Brciman L in 2001 as an integrated learning method for classification, regression, and other tasks that builds a forest of decision trees (a collection of decision trees) using a Bootstrap Aggregating (bagging) method and outputs the average of the predictions of individual decision trees [48].

#### 2.5.2. Optimizing Parameters 

The hyperparameters of RF regression include n_estimators, max_depth, min_samples_split, min_samples_leaf, and so on. Optimizing the hyperparameters of a random forest can help us find the best model configuration and improve the predictive accuracy and robustness of the model. By adjusting the hyperparameters, the complexity of the model can be effectively controlled to avoid overfitting or underfitting phenomena. Thus, optimizing the hyperparameters of a random forest regression model is crucial to improve the performance of the model and to make it more applicable to different datasets and problem domains.

The bald eagle search (BES) optimization algorithm [49] is a population intelligence optimization algorithm that simulates the behavior of bald eagles in capturing prey which was proposed by Alsattar et al. in 2020 and has received wide attention since its proposal.

The BES algorithm has the performance advantages of fast convergence speed and simple structure, and its principle is to simulate the behavior of a condor preying on its prey. The algorithm can be divided into the three phases of selecting the search space, searching for the prey, and swooping down to capture the prey. The mathematical model is shown below:

1. Selection of search space: Within the selected search space, the bald eagle identifies and determines the optimal search area by judging the amount of prey, which facilitates the capture of prey. The specific mathematical model of this behavior is described as:

(8) $$P_{i,\mathrm{new}}=P_{\mathrm{best}\;}+\alpha \;\times \;r\;\left(P_{\mathrm{mean}}-P_{i}\right)$$

where α denotes the control position change parameter with a value in the range of (1.5, 2) and r is a random number with a value in the range of (0, 1); P_i,new_ is the new best search area position determined by the ith bald eagle, which is determined by randomly searching for the previous information multiplied by α to determine it, which is used to randomly change all search points; P_best_ is the current best search area location; P_mean_ is the average of the search area locations of all the bald eagles at the end of the previous searches; P_i_ is the ith bald eagle location.

2. Searching for prey: Bald eagles adopt a characteristic spiral shape of flight for searching, and mathematical models are developed to represent this movement using polar coordinate equations.

(9) $$\theta \left(i\right)=a\;\times \pi \;\times \;\mathrm{rand}\;$$

(10) $$r\left(i\right)=\theta \left(i\right)+R\times \mathrm{rand}\;$$

(11) $$\mathrm{xr}\left(i\right)=r\left(i\right)\times \sin\left(\theta \left(i\right)\right)$$

(12) $$\mathrm{yr}\left(i\right)=r\left(i\right)\times \cos\left(\theta \left(i\right)\right)$$

(13) $$x\left(i\right)=\mathrm{xr}\left(i\right)/\max\left(\left|\mathrm{xr}\right|\right)$$

(14) $$y\left(i\right)=\mathrm{yr}\left(i\right)/\max\left(\left|\mathrm{yr}\right|\right)$$

where θ(i) and r(i) are the polar angles and polar diameters of the helix equation, respectively; a and R are the parameters controlling the helix trajectory, where a is used to determine the angle between the search points at the center point (which takes the value range of (5, 10)) and R is used to determine the number of search cycles (which are in the value range of (0.5, 2)); rand is a random number in the value range of (0, 1); and x(i) and y(i) denote the position of the bald eagle in polar coordinates, both in the value range of (−1, 1).

Bald eagles search for prey within a selected search space and move in different directions within the spiral space, thus accelerating the search. The optimal position of the dive can be expressed by the following equation, where P_i+1_ is the next update position of the ith bald eagle.

(15) $$P_{i,\mathrm{new}}=P_{i}+x\left(i\right)\times \left(P_{i}\;-\;P_{\mathrm{mean}\;}\right)+y\left(i\right)\times \left(P_{i}-P_{i+1}\right)$$

3. Swooping to capture prey: Bald eagles quickly swoop and fly towards the target prey from the best position in the search space, and other individuals of the population also move towards the best position and attack the prey at the same time. The polar coordinate equation is still used to establish the mathematical model:

(16) $$\theta \left(i\right)=a\times \pi \times \mathrm{rand}\;$$

(17) $$r\left(i\right)=\theta \left(i\right)$$

(18) $$\mathrm{xr}\left(i\right)=r\left(i\right)\times \sin h\left[\theta \left(i\right)\right]$$

(19) $$\mathrm{yr}\left(i\right)=r\left(i\right)\times \cos h\left[\theta \left(i\right)\right]$$

(20) $$x\prime \left(i\right)=\mathrm{xr}\left(i\right)/\max\left(\left|\mathrm{xr}\right|\right)$$

(21) $$y\prime \left(i\right)=\mathrm{yr}\left(i\right)/\max\left(\left|\mathrm{yr}\right|\right)$$

The formula for updating the bald eagle position during a dive is:

(22) $$P_{i,\mathrm{new}}=\mathrm{rand}\;\times \;P_{\mathrm{best}}+x\prime \left(i\right)\;\times \;\left(P_{i}-c_{1}\;\times \;P_{\mathrm{mean}\;}\right)+y\prime \left(i\right)\;\times \;\left(P_{i}-c_{2}\times P_{\mathrm{best}}\right)$$

where c_1_ and c_2_ increase the intensity of the vulture’s movement towards the optimum and the center point, taking values in the range of [1,2].

The BES algorithm finds the optimal or near-optimal solution of the optimization problem by iteratively searching for the optimal way, using the continuous updating of the positions of the bald eagle individuals in the search space. The input parameters of the BES algorithm consist of population size, maximum number of iterations, upper and lower bounds of the search space, problem dimensionality, and fitness function. The first few of the parameters are selected based on the problem’s complexity and the computational resources available. The fitness function is an effective measure for evaluating the strengths and weaknesses of the problem to be optimized, which can relate the BES algorithim to the RF algorithm. Therefore, the position for the individual vulture with the optimal value of fitness can be considered as the best solution for the optimization problem.

The fitness function is designed as follows:

- Training a random forest model based on the optimized combination of parameters;
- Using the trained model to predict both the training set and the test set, the prediction results obtained are T_sim_1 and T_sim_2;
- Calculating the mean squared error (MSE) between the prediction results and the true values separately, and then taking the sum as the fitness value.

**Figure 10:**
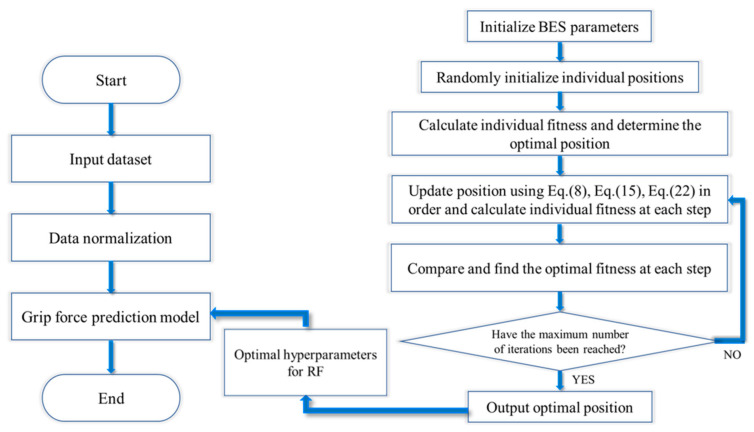
BES algorithm flowchart.

By optimizing the combination of parameters for n_estimators and min_samples_leaf through the BES algorithm, the fitness value is minimized, thus obtaining the optimal bald eagle individual position, which is the best-performing parameter combination in model training, thereby optimizing the performance of the random forest model. The algorithm terminates when it reaches the maximum number of iterations and outputs the optimal solution at that point. The algorithm procedure of the BES−RF model is shown in Figure 10.

#### 2.5.3. Model Evaluation Methods 

There are many evaluation metrics for predictive modeling. Xue et al. uses mean squared error (MSE), mean absolute error (MAE) and R-square (R^2^) as evaluation metrics [45]. Mao et al. uses R^2^, MAE, etc. [50]. Chicco et al. noted that R^2^ shows higher reliability in reflecting the effect of regression prediction models compared to other performance indicators [51]. Therefore, in this paper, MAE, MSE, and R^2^ are used as the evaluation indicators of the model in order to comprehensively evaluate the performance of the model. These metrics are defined by Equations (23)–(25) in turn.

(23) $$\mathrm{MAE}=\frac{1}{N}\sum_{i=1}^{N}\left|\left(f_{i}\;-\;f_{i-p}\right)\right|$$

(24) $$\mathrm{MSE}=\frac{1}{N}\sum_{i=1}^{N}{\left(f_{i}\;-\;f_{i-p}\right)}^{2}$$

(25) $$R^{2}=1-\frac{\sum {\left(f_{i-p}-f_{i}\right)}^{2}}{\sum {\left(f_{i}\;-\;f_{i,\mathrm{mean}}\right)}^{2}}$$

f_i_ represents the true grip force, f_i−mean_ represents the average value of grip force, f_i−p_ represents the force predicted by the model based on the sEMG signal, and N represents the total number of samples. 

MAE can visualize the difference between f_i_ and f_i−p_. MSE is more sensitive to outliers, facilitating model convergence. R^2^ can accurately reflect predictive performance, ranging between 0 and 1. R^2^ is closer to 1, indicating superior effectiveness.

## 3. Results

### 3.1. Test of sEMG Acquisition System

#### 3.1.1. Circuit Parameters Test of sEMG Acquisition Terminal 

A number of circuit parameters were tested, and the results are shown in Table 1. Among them, the CMRR performance was excellent. It was better than [34], when the input frequency was 100 Hz, and it can still maintain more than 80 dB when the input signal frequency reached 500 Hz, which can meet the high CMRR requirements in the frequency range of sEMG. A 3.7 V, 500 mAh Li-ion battery was selected to supply power. The terminal can be used for up to 6–7 h, which meets the demand for continuous monitoring.

#### 3.1.2. Function Test of System 

The functional reliability of the system designed in this paper was verified in the following two ways:

- Comparing the sEMG signals acquired between this system and a generic wireless sEMG signal acquisition system available on the market;
- Comparing the sEMG signals for different hand movements acquired by this system.

Both of all were performed based on the ECRL muscle.

The Delsys wireless EMG signal acquisition system has been widely used in various studies [29,52,53], and can be considered as a standard. Due to the small size of the ECRL muscle, it is not possible to simultaneously attach two sensors at the same location for simultaneous acquisition (the system proposed in this manuscript as well as the Delsys system). As a result, two acquisition experiments were performed using two sensors separately. The skin surface on which the electrodes were placed was permanently marked using a marker pen to ensure the sensor attachment location was the same in both experiments [54]. Normalization of the signals afterwards is necessary because of the difference between output ranges of each system. The utilization of disparate electrode designs, electrode spacing, and other variables may result in subtle variations in the raw sEMG signals acquired.

**Figure 11:**
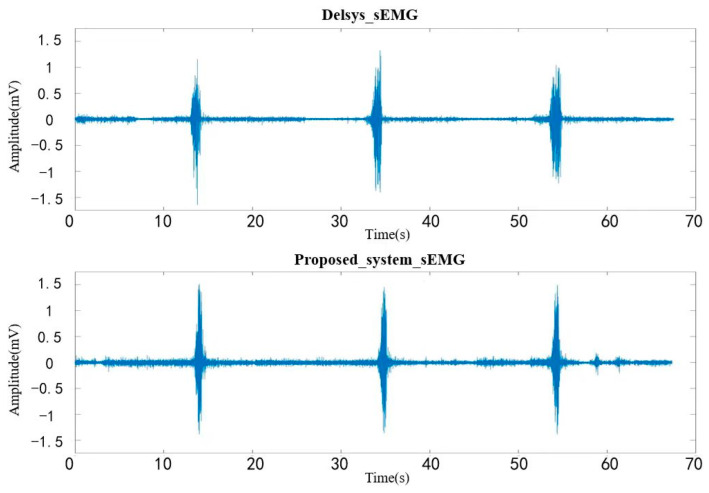
Comparation of the raw sEMG signals acquired by Delsys system and proposed system in three maximum grip force experiment.

**Figure 12:**
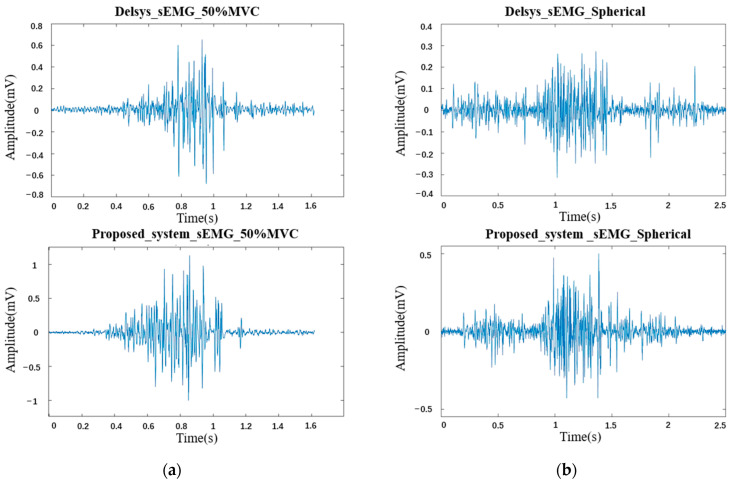
Comparation of the raw sEMG signals acquired by Delsys system and proposed system: (**a**) 50% MVC grasping and (**b**) spherical grasping.

Three maximum grip force experiment was first performed for each of the two sensors, and the MVC based on each sensor was recorded by taking the mean. Secondly, the sensor measurements were taken in a randomized order, and the sEMG signals were recorded on five separate occasions, while the subjects performed a 50% MVC grasping as well as a spherical grasping movement. A 5 min rest period was guaranteed between the two sets of measurements; each movement had a 20 s muscle firing interval during the measurements to prevent muscle fatigue. The sEMG signals were segmented, filtered, rectified, normalized and envelope smoothed after the acquisition was completed, and then the waveform data of the two experiments were aligned. Raw sEMG signals of the two systems for the three maximum grip force experiments is shown in Figure 11 and Figure 12, illustrating the raw sEMG signals for both systems when performing a 50% MVC grasping as well as a spherical grasping.

Cross-correlation coefficient (CCC) was selected as the evaluation parameter [55]. CCC is a widely used method for comparing EMG signals [56] and can be kept between −1 and 1, where −1 indicates negative correlation and 1 indicates positive correlation. The formula is as follows:

(26) $$\mathrm{CCC}=\frac{n\left(\sum xy\right)\;-\;\left(\sum x\right)\left(\sum y\right)}{\sqrt{\left[n\left(\sum x^{2}\right)-{\left(\sum x\right)}^{2}\right]\left[n\left(\sum y^{2}\right)-{\left(\sum y\right)}^{2}\right]}}$$

The results are displayed in Table 2. The maximum, minimum, mean, and standard deviation of the CCC are included.

In addition, the RMS characteristics corresponding to the processed waveforms of the two systems are derived separately and the agreement (1 − RE) is calculated, which is basically more than 90%. Six groups were randomly selected and shown in Table 3.

(27) $$1\;-\;\mathrm{RE}=(1\;-\frac{\left|x\;-\;y\right|}{\left|y\right|})\times 100\%$$

These results indicated a good agreement between the proposed system and the standard system.

**Figure 13:**
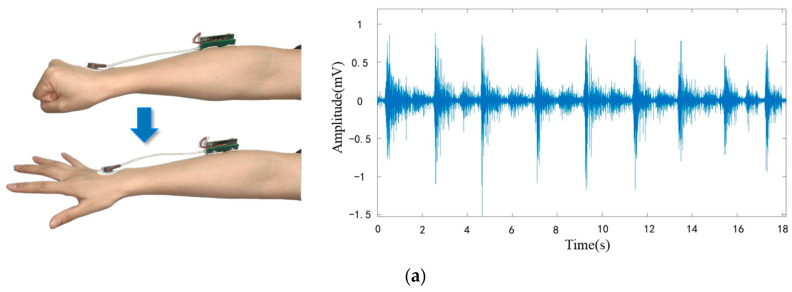

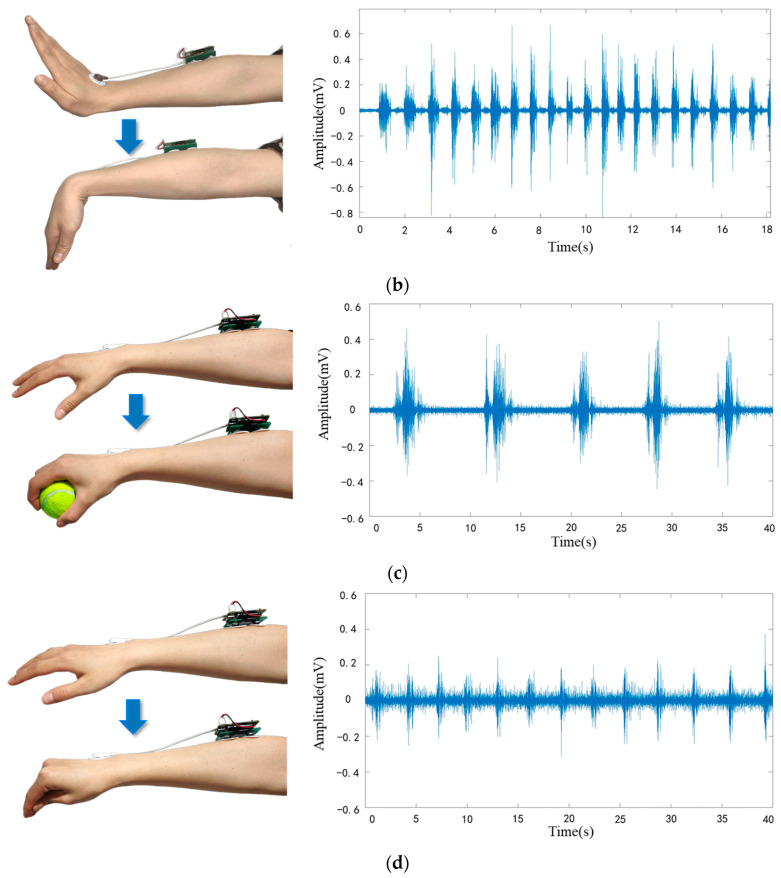
The sEMG signals at two different movement: (**a**) continuous clenching to unclenching of the fist; (**b**) continuous up and down turning of the wrist; (**c**) continuous spherical grasping and (**d**) continuous finger pinch.

As shown in Figure 13, there are four movement variations, including (a) continuous clenching to unclenching of the fist, (b) continuous up and down turning of the wrist, (c) spherical grasping, and (d) finger pinch. sEMG signals of both movements were acquired. It can be found that this system can capture the EMG signals of different hand movements.

### 3.2. Test of Algorithm 

More than 200 data points were collected for the experiment. The experiment used 70% of the data as a training set and 30% as a test set. Firstly, the parameters of the BES algorithm were adjusted to explore the effect of parameter settings of the BES algorithm on the overall algorithm. Table 4 displays the evaluation metrics of the BES−RF algorithm with various parameters. The results suggest that the model performs optimally when the population size is 10 and the maximum number of iterations is 30.

The population size is the number of solutions considered simultaneously in the optimization process. In the given results, the model performs better with a population size of 10, which may be due to the fact that 10 candidate solutions can already adequately cover the solution space while keeping the computational cost low. The maximum number of iterations determines the total number of iterations rounds for which the vulture optimization algorithm is run, which affects the running time and convergence speed. In the given result, it may be because 30 iterations are enough for the algorithm to converge to the optimal solution while avoiding overfitting. The optimal combination of parameters in the results may strike a good balance between exploratory power and computational efficiency. However, the optimal values of these parameters may vary depending on the problem and dataset, so, in practice, experimentation and tuning are still needed to determine the optimal parameter combinations.

Secondly, the BES−RF algorithm is compared with RF, artificial neural network (ANN), and polynomial regression (PR) algorithms. The results are as shown in Table 5.

By comparing several algorithms commonly used in force prediction, we find that BES−RF and RF have better performance compared to the other two algorithms. sEMG signals may have a complex nonlinear relationship with grip force [Surface EMG in advanced hand prosthetics], and the Random Forest algorithm is based on the integrated learning method of decision trees. The Random Forest algorithm, which is based on the integrated learning method of decision tree, is able to capture and model this nonlinear relationship well and is robust to noise and outliers. Additionally, it has a lower risk of overfitting than ANN and polynomial regression, so it demonstrates a better performance in this experiment. Secondly, we can also find that the BES algorithm improves the performance of the RF prediction model, and the R^2^, MAE, and MSE of BES−RF are all improved.

## 4. Discussions

The multi-channel wireless acquisition system developed in this paper is portable and wearable, with upper computer software that can display waveforms in real-time, is easy to operate, and has a quality of signal acquisition that is in good agreement with commercial devices. A grip force prediction model was established by using the BES−RF algorithm.

A VR-based sEMG-triggered grip strength exercise platform that quantitatively grades grip force by measuring sEMG signals has been developed [28]. However, it does not accurately predict the magnitude of grip force values. In this case, the sEMG was acquired using a commercial device, Biopac MP150 (Biopac Systems Inc.). The work of [29] is based on the Delsys system. Both commercial devices are expensive, while the proposed system reduces the system cost by choosing cost-effective components and optimizing the PCB layout.

Table 6 demonstrates the comparison between this work and other research work. In the wireless transmission system, the proposed four-channel discrete acquisition terminal is more flexible and can better monitor the interactions and influences between muscles at different locations than the integrated acquisition terminal [30,57]. The proposed system is a system not limited by the wiring between the acquisition electrodes and the processing circuitry. Connecting the electrodes by snap fasteners to reduce wire length and suppressing interference by symmetrical differential signals makes the system more adaptable to the environment with high common mode rejection ratios across the main frequency range of sEMG.

Increasing the number of channels usually improves prediction accuracy. The weight of the single-channel sEMG acquisition module designed for this study is 31.80 g. If the number of channels in a single forearm is increased from 2 to 4, the increase in the number of channels results in an increase in weight, which may be more burdensome for the subjects, so the size and weight of the sensor needs to be reduced, e.g., by using an integrated analogue front-end chip to reduce the area of the circuitry, making it less burdensome for the subject in the case of four channels. 

At the same time, as the number of channels increases, it is necessary to select appropriate electrode positions to reduce inter-muscle crosstalk and ensure accurate signal acquisition. The degree of crosstalk can be determined by selecting different muscle combinations and performing cross-correlation analysis, crosstalk risk factor (CRF) analysis, and co-activation confidence (CCA) analysis [58] under a specific exercise mode (e.g., grip strength), which can be used to guide the electrode placement strategy. Ultimately, muscles that are less susceptible to crosstalk will be selected for the study. In addition, researchers are investigating methods to improve prediction accuracy when the number of channels is reduced, e.g., a deep neural prediction network with a one-dimensional convolutional structure achieves high prediction accuracy with as few channels as possible [45].

In this study, the wrist was chosen as the reference electrode location because it is far away from the target muscle and suffers less interference. In addition, in cases of special circumstances, such as scarring or oedema in the skin of some subjects, the location of the reference electrode needs to be individually adjusted and optimized to ensure good contact between the electrode and the skin, and other bony prominences or electrically neutral locations can be chosen, such as the olecranon (elbow) [59].

The current study has implemented grip force prediction for standard movement in an offline state. In order to realize applications in continuous grip strength monitoring, the trained (RF) model for offline prediction can be combined with online learning for real-time model updating [60] using an online hyperparameter tuning approach to dynamically adjust RF hyperparameters [61]. After receiving the data on the PC side, the real-time overlapping sliding window processing algorithm [44] will be used to process and extract features from the sEMG under each window in real-time, and these features are input into the RF model after online learning to predict the grip force in real-time. Meanwhile, the computational efficiency and memory consumption of the algorithm need to be further solved to meet the real-time requirements of online learning. 

Our proposed system, designed as a muscle status monitoring tool, has potential applications in the rehabilitation of patients and the daily training for healthy individuals. For these applications, the current acquisition experimental steps of the system include attaching an electric electrode device to a fixed muscle and opening the system interface for signal acquisition. The small size of the device makes it portable, and the steps are relatively easy to use. In addition, the user range will be continually expanded by experimentally guiding the optimizations of hardware and algorithms. For force prediction under other training movements, there are more studies based on sEMG to classify multiple fine-training movements for stroke patients [62], and force prediction under these fine movements will be our future research direction.

## 5. Conclusions

This paper proposes and designs a portable, wearable, low-cost, and easy to operate multi-channel sEMG wireless acquisition system for muscle status monitoring. An sEMG acquisition terminal can achieve a gain of 1125×, a CMRR of 84.12 dB at 500 Hz, a filter bandwidth of 15.4 Hz to 1.86 kHz, and a sampling rate of 1000 Hz. Then, sEMG signals and grip force were acquired using this system in combination with a grip force meter. Subsequently, sEMG was pre-processed and feature extracted to build a grip force prediction model. The model used the BES algorithm to optimize the hyperparameters of RF, which improved the performance of the prediction model. The best result by the BES−RF model was as follows: R^2^ = 0.9215, MAE = 1.0637, and MSE = 1.7479. This model can be used for predicting hand grip force and for real-time prediction of grip force in future studies. The current study is focused on monitoring muscle force, and additional muscle states will be investigated in the future, aiming to provide a more convenient and effective monitoring tool for rehabilitation of hand function and disease condition monitoring in patients, as well as scientific exercise in healthy individuals. 

The future aims are as follows: adapting the circuit design to reduce the size and weight of the device and make it more suitable for sensation without monitoring; using a wider bandwidth for an sEMG signal of 5 to 500 Hz to capture the frequency of motor unit discharges as well as physiological tremors more comprehensively; optimizing the BES−RF algorithm to improve prediction accuracy and real-time online grip force prediction; conducting clinical trials in stroke patients and using clinical scales such as the ARAT to more accurately assess patients’ functional recovery and explore the potential application of the grip force transformation ability in the stroke population; conducting further research into multiple muscle states, such as muscle fatigue.

## Figures and Tables

**Table 1:** Circuit parameters of sEMG acquisition terminal.

Operating Voltage	Reference Voltage	Sampling Rate	Gain	CMRR	Filter Bandwidth
3.3 V	1.65 V	1000 Hz	1125	84.12 dB (500 Hz) 96.94 dB (100 Hz)	15.4 Hz–1.86 kHz

**Table 2:** The maximum, minimum, mean, and standard deviation of the CCC.

	50%MVC Grasping	Spherical Grasping
CCC_max	0.9890	0.9643
CCC_min	0.8853	0.8388
CCC_mean	0.9229	0.9132
Standard deviation	0.03	0.05

**Table 3:** RMS characteristics and 1 − RE of six groups.

Movement	Name	RMS1	RMS2	RMS3	RMS4	RMS5	RMS6
50%MVC grasping	Delsys	0.0486	0.0623	0.0498	0.0572	0.0463	0.0667
	Proposed 1 − RE	0.0535 89.92%	0.0596 95.67%	0.0486 97.6%	0.0589 97.03%	0.0502 91.58%	0.0630 94.46%
Spherical	Delsys	0.0379	0.0594	0.0353	0.0467	0.0299	0.0526
	Proposed 1 − RE	0.0371 97.89%	0.0621 95.46%	0.0384 91.22%	0.0443 94.87%	0.0332 88.96%	0.0486 92.4%

**Table 4:** Evaluation metrics of the BES−RF algorithm with various parameters.

Population Size	Maximum Number of Iterations	R^2^	MAE	MSE
10	10	0.8926	1.0873	1.8738
10	20	0.8883	1.2365	2.167
10	30	0.9215	1.0637	1.7479
10	40	0.9012	1.1113	1.8027
20	30	0.8988	1.2215	2.1537
30	30	0.8904	1.2031	2.0248

**Table 5:** Evaluation metrics of different algorithms.

Algorithms	R^2^	MAE	MSE
BES−RF	0.9215	1.0637	1.7479
RF	0.8792	1.3475	2.1938
ANN	0.8769	1.4039	2.1899
PR	0.8699	1.6729	2.2674

**Table 6:** The comparison with other works.

Research	Channel	Portable	Wearable	Flexibility	Application	Method
[31]	3	NO	NO	Poor	Lower limb Muscle force estimation	GA-SVR algorithm
[33]	8	NO	NO	Poor	Grip force estimation	EMG imaging
[34]	8	YES	YES	Good	Real-time acquisition	-
[57]	8	YES	YES	Moderate	Fatigue monitoring	SVM algorithm
[30]	2	YES	YES	Moderate	Real-time acquisition/ monitoring	RMS Calculation
Proposed	4	YES	YES	Good	Grip force prediction	BES−RF algorithm

## Data Availability

The raw/processed data required to reproduce these findings cannot be shared at this time as the data also form part of an ongoing study.
